# Crested macaque facial movements are more intense and stereotyped in potentially risky social interactions

**DOI:** 10.1098/rstb.2021.0307

**Published:** 2022-09-26

**Authors:** Peter R. Clark, Bridget M. Waller, Muhammad Agil, Jerome Micheletta

**Affiliations:** ^1^ Evolution and Social Interaction Research Group, Nottingham Trent University, Nottingham NG1 4FQ, UK; ^2^ Centre for Comparative and Evolutionary Psychology, University of Portsmouth, Portsmouth PO1 2UP, UK; ^3^ Macaca Nigra Project, Tangkoko-Batuangus Nature Reserve, North Sulawesi, Indonesia; ^4^ Faculty of Veterinary Medicine, Agricultural University of Bogor, Bogor, Jawa Barat 16680, Indonesia

**Keywords:** primates, facial expressions, communication, FACS

## Abstract

Ambiguity in communicative signals may lead to misunderstandings and thus reduce the effectiveness of communication, especially in unpredictable interactions such as between closely matched rivals or those with a weak social bond. Therefore, signals used in these circumstances should be less ambiguous, more stereotyped and more intense. To test this prediction, we measured facial movements of crested macaques (*Macaca nigra*) during spontaneous social interaction, using the Facial Action Coding System for macaques (MaqFACS). We used linear mixed models to assess whether facial movement intensity and variability varied according to the interaction outcome, the individuals' dominance relationship and their social bond. Movements were least intense and most variable in affiliative contexts, and more intense in interactions between individuals who were closely matched in terms of dominance rating. We found no effect of social bond strength. Our findings provide evidence for a reduction in ambiguity of facial behaviour in risky social situations but do not demonstrate any mitigating effect of social relationship quality. The results indicate that the ability to modify communicative signals may play an important role in navigating complex primate social interactions.

This article is part of the theme issue ‘Cognition, communication and social bonds in primates’.

## 1. Background

Given the importance of communication for forming and maintaining social bonds, failures in communicative signalling can be costly to the signaller and/or receiver. The potential for fitness costs means that there is selective pressure on communicative signals to be clear and unambiguous ([1], p.882; [2]). However, some signals might be under more selective pressure than others. Signals that are used for different functions, by different animals, and in different environments are under different levels of evolutionary pressure; misunderstanding an alarm call could cause fatal results, but misunderstanding a contact call probably won't. Understanding the links between signal characteristics and their different functions within the formation and maintenance of social bonds is therefore important for our understanding of the evolution of complex signals, and the conditions that give rise to flexibility.

Given the possibility of costly communication failures, signals should be less ambiguous in situations where environmental or social factors mean that there is a high chance of misunderstandings. Misunderstandings can occur for two reasons: ineffective signal transmission or misinterpretation. Signal transmission problems occur when the signal does not pass easily through the environment; for example, female great tits respond more slowly to their mates' calls in noisier environments [3]. Misinterpretation of a signal is more likely in situations where the individuals involved are unfamiliar with each other, as has been demonstrated in human studies of interactions with friends and strangers (e.g. [4,5]). Ambiguity should also be reduced where misunderstandings would be very costly, such as in the case of predator alarms, aggressive interactions and mating calls.

There are many examples of reductions in ambiguity of signals in situations where misunderstandings are likely, with signals often becoming more intense, more ritualized (i.e. less variable) and more specific to their function. For example, to ensure effective communication in noisy environments many birds, including male great tits, increase the volume and pitch of their songs, making the signals more intense [3,6]. North American wren species inhabiting areas of dense conspecific population and sparse heterospecific population (i.e. areas where the probability of encountering a conspecific is greater than that of encountering a different species) have more diverse song repertoires than species inhabiting areas with sparse conspecific population and dense heterospecific population [7]. In the latter species, song variability is constrained by the need to reliably identify members of the same species in the more demanding social landscape, and so individual songs might contain fewer sequences, be more repetitive, and be louder. Furthermore, alarm calls of many species reduce ambiguity with specificity and intensity. Vervet monkeys (*Chlorocebus pygerythrus*) exhibit distinct calls when they sight a leopard, eagle or snake [8], while meerkats and prairie dogs each have very complex alarm call repertoires where calls are tailored to a specific threat, encoding information about not only the type of predator but also how far away it is and whether it is moving [9–11]. Courtship rituals often involve intense energetic displays and combinations of multiple signal modalities (e.g. widowbirds [12], sage grouse [13]) and male–male competition often involves intense displays (e.g. red deer roars [14]).

In sum, there is a great deal of evidence for increased intensity and specificity of communicative signals that are used (1) for more immediately important functions, such as mating and survival and (2) in conditions that are susceptible to uncertainty and high costs of failure. However, non-human primates can also use signals flexibly; for example, great apes with small vocal repertoires use calls in a variety of contexts, with outcomes less tightly tied to the signals themselves but rather to the context, in terms of both the immediate behaviour accompanying the signal and the pre-existing relationship between the signaller and receiver [15]. In this example, it could be argued that the signal is under less constraint because the pre-existing relationship between the two individuals and the cognitive ability of each individual are strong enough for misunderstandings to be less likely (and potentially less costly).

By contrast to the many studies of animal vocalizations, facial behaviour of non-human primates has attracted relatively little attention. Facial movements are prominent in many social interactions of non-human primates, and descriptive work has identified some common facial configurations that persist across many species (e.g. [16]). However, while this representation of facial behaviour implies discrete, static configurations and facial ‘expressions’ have been treated as such in many studies, this does not reflect the reality of facial displays in many species (e.g. crested macaque [17], Tonkean macaque [18]). While some descriptions of facial displays document the presence of dynamic displays (e.g. [19]), these are also defined by their rigidity; deviation from a ritualized template is an argument for not considering a display to be part of the repertoire. For example, it is for this reason that some researchers do not consider teeth-chattering a separate display, instead describing it ‘rapid alternation between silent bared-teeth and lipsmack’ [19].

As well as differing in specific identifying features such as the presence of characteristic movement or combination of movements, facial behaviour often differs in general features such as duration, intensity and variability. Inter- and intra-individual differences in the intensity and variability of facial movements have not been studied in detail in non-human animals; behavioural ecologists focus instead on repertoires exhibited by species as a whole (e.g. [20–22]). The Facial Action Coding System (FACS; [23,24]) allows more objective measurement of facial movements, based on the underlying muscle contractions. However, despite the advantage of more objective methods, very few studies use FACS to examine facial movements of human subjects, and even fewer for non-humans. Perhaps due to the time-consuming nature of FACS coding [25], coding of spontaneous facial movements in humans and non-humans tends to focus on only the ‘peak’ activation (e.g. [26,27]) rather than the variability of movements produced throughout an interaction (but see for example [17,28]). Adaptations of FACS to nonhuman primates (e.g. ChimpFACS [27], MaqFACS [29]) do not generally include guidance for coding intensity of action units (AUs), and studies using FACS in non-humans tend to concentrate on comparison of AU combinations between contexts and/or species rather than on the variability or intensity of facial movements in single interactions. In non-human primates, only one study has so far examined the intensity of AUs [17], finding reduced intensity in bared-teeth displays associated with affiliation compared to displays associated with copulation or submission. The same study found that crested macaque bared-teeth displays were more variable in affiliative contexts than in play, submission and copulation contexts. One study of play in gorillas that did not use FACS methodology or measure intensity of single movements found that more intense play bouts involved higher rates of lip withdrawal [30], which would appear to tally with our expectation of more intense signals in situations where ambiguity of signals would be costly. Wider research has concentrated on quantifying the variability of facial communication systems by building repertoires of AUs for entire species (e.g. [20,22]), rather than examining in high temporal resolution the variability of movements involved in single interactions. With FACS, variability of movements can be quantified in various ways, including the repertoire of AUs (e.g. [20]), the diversity of AUs produced (e.g. [22]), the repertoire of AU *combinations* (e.g. [22]) and the rate of change of AU combination (e.g. [17,28]). Each of these measures may give subtly different insights into the facial behaviour of the subject. Since crested macaques also produce dynamic ritualized facial displays [19], such as lipsmacking [31], differences in the rates of these will also illustrate the variability of facial movements.

In this study we investigated whether general characteristics of crested macaque facial movements are affected by (i) the behaviour exhibited in the present social interaction and (ii) the long-term social relationship history of the animals involved. Crested macaques live in multi-male, multi-female groups, and while generally dominance hierarchies are well-established and linear, their social style is relatively tolerant compared to other macaques; low-intensity conflict (i.e. without physical contact) is common but serious fights are rare [32], and there are high rates of counter-aggression and post-conflict reconciliation [33,34]. We expect this relatively complex social environment to be associated with relatively complex communication [35], which is likely to involve differences in intensity and variability of facial signals. First, we expected that higher intensity and lower variability are used in more risky situations such as aggression and submission, with lower intensity and greater variability in affiliative contexts. This has already been demonstrated to an extent with silent bared-teeth displays [17], and would be expected to continue for all facial behaviour given the greater selection pressure of high-cost situations such as conflicts.

Second, we expected that facial movements would be more intense and less variable when (1) directed up the dominance hierarchy, (2) used between individuals who are close in dominance rank and (3) used between individuals sharing a weak social bond. These expectations are because (1) costs are likely to be lower for dominant individuals, (2) there is a reduced likelihood of conflict when dominance rank is very different and (3) there is less chance of misunderstandings occurring between individuals that know each other better.

## 2. Methods

### (a) Data collection

Data were collected between December 2018 and April 2019 at the Macaca Nigra Project (MNP; www.macaca-nigra.org) field site in Tangkoko-Batuangus Nature Reserve, North Sulawesi, Indonesia (study site described in detail in [36,37]). At that site, we followed members of two different social groups, conducting focal follows of known adult individuals [38]. In one group (PB1b) we followed all adults, and due to the female-biased sex ratio of this group we also followed all adult males in a second group (R2) in order to get a more representative sample of expressions produced by males. Full details of the groups and the individuals followed are shown in table 1. In focal observations, two observers followed an individual at a distance of 3–20 m, depending on terrain, vegetation, and the location of the individual within the group. One observer kept a video camera (Panasonic HDC-SD700, Bracknell, UK) trained on the animal's face and activated the pre-record function on the camera whenever a facial movement was produced and whenever another individual approached or was approached by the focal animal. The other observer used a tablet computer with a purpose-built macro in Microsoft Excel to collect continuous behavioural data, including general activity (move, rest, forage etc.) and detailed records of social interactions, and scan data (every five minutes) including identity of any other individuals within 1 m and 5 m. Since both camera and tablet recorded timestamps of recordings, it was possible to match video clips with social interactions recorded in the focal files. In addition to the video footage obtained from focal animals, we captured some video footage of facial movements produced by non-focal animals, either when the non-focal animal interacted with the focal animal being followed, or opportunistically in between focals.

**Table 1.** Characteristics of the groups followed during data collection. N.B. As juveniles and infants are not individually recognizable, their numbers are approximate, as are total group sizes. Numbers of subadult males changed throughout the study period; numbers given represent most common situation.

	group	
	R2	PB1b
adult males		
individuals present	6	4
individuals followed (for at least 5 h)	5	4
adult females		
individuals present	14	20
individuals followed (at least 5 h)	0	17
subadult males		
individuals present	4	3
individuals followed (at least 5 h)	1	1
juveniles + infants present	c.15	c.25
total group size	c.41	c.52

### (b) Coding behavioural outcome

By matching video clips to the behavioural data, we were able to categorize the clips based on the behaviour that immediately followed the production of a facial movement; the categories were affiliation, copulation, submission and aggression (see electronic supplementary material, table S1 for behaviours in each category). If facial movements were produced while social behaviour was ongoing, we categorized these based on the accompanying behaviour, as long as that behaviour continued beyond the point when the facial movements stopped. Some video footage showed interactions that included distinct phases, such as aggression followed by reconciliation; in these cases, we split the footage into two or more clips so that facial behaviour produced before aggressive acts was analysed separately from that produced before affiliative behaviours.

During data cleansing, we adjudged whether clips were of sufficient quality to enable FACS coding, discarding clips where visibility of the face was inadequate, for example due to distance, poor focus or presence of obstructions. From a total of 1545 video clips collected, this yielded a total of 506 codable videos. In 142 of these, the signaller or receiver was unknown, meaning dyadic information was not available. The remaining 364 clips, showing facial movements of 35 different individuals in total, were FACS coded and analysed. Each clip showed a single interaction, in which both signaller and receiver were individually recognizable adults or subadults, enabling the calculation of standard measures of dominance relationship and social bonds (detailed below).

### (c) FACS coding

Video clips were coded using BORIS [39] by a certified MaqFACS coder (P.C.) following the guidelines of MaqFACS [29], which are applicable to crested macaques with minor alterations [17]. To minimize the coder's knowledge of the context of each video clip, the clips were trimmed to cover only the period during which facial movements were observed. We coded whether the focal animal was looking at the target individual—based on the direction of the face during the majority of facial movements—and analysed only the movements produced when either (a) the animal was looking at the target individual or (b) the animal was in body contact with the target individual and not looking at a second individual. This rule was devised to distinguish those cases where an individual looked away from the original target in order to direct facial signals toward a second individual from those where the change in gaze direction was irrelevant to the continuation of the communicative bout.

### (d) Calculating facial movement measures

For each video clip, we calculated the overall intensity of facial movements. This was based on only the AUs for which coding intensity was possible: AU10 (upper lip raiser), AU12 (lip corner puller), AU26 (jaw drop) and EAU3 (ear flattener). Unlike the other AUs, these movements were conspicuous enough that we could tell the difference between major and minor movements, and coding of intensities for these AUs has been shown to be reliable previously [17]. These four AUs are very common, being used in various displays described by van Hooff [16]: silent bared-teeth (AU10 + 12 + 26 + EAU3), staring open-mouth (AU26 + EAU3), staring/frowning bared-teeth scream (AU10 + 12 + 26 + EAU3), lip-smacking (AU26 + EAU3), teeth-chattering (AU10 + 12 + 26 + EAU3) and relaxed open-mouth (AU10 + 12 + 26). These displays are associated with various social interaction types, covering the four umbrellas of affiliation, aggression, copulation and submission. In our dataset, at least one of these AUs was present in every interaction, and most interactions involved activation of at least two of these AUs (see electronic supplementary material, table S2). Each of these individual AUs was active in at least 39% of interactions for each outcome category, meaning that none of these AUs was particularly associated with a single interaction outcome or function (see electronic supplementary material, table S3).

Overall intensity of movements was calculated for each video using the following formula:

$$\mathrm{intensity}=\frac{H_{10}+H_{12}+H_{26}+H_{EAU3}+T_{27}}{T_{10}+T_{12}+T_{26}+T_{EAU3}+T_{27}}$$

where $H_{n}$ is the duration of time that action unit *n* was produced at high intensity and $T_{n}$ is the total duration of action unit *n.* While we did not code intensity of AU27 (jaw stretch), it can be thought of as an exaggerated version of AU26 (jaw drop), so was treated as a major AU26 for the purpose of this calculation. Since every interaction involved at least one AU that was coded for intensity (electronic supplementary material, table S2), this formula yielded a value between 0 and 1 for every interaction.

Variability was calculated using two components. First, unpredictable variability was calculated as the number of changes in AU combination, plus the number of changes in intensity of AU10, AU12, AU26 and EAU3, divided by the length of the video in seconds. Second, predictable variability was the number of frames for which a repetitive dynamic action descriptor (RDAD)—AD181 (lipsmack), AD182 (teeth chatter), AD183 (tongue chatter), AD184 (jaw wobble)—was active, divided by the overall duration of the facial movements in frames, and multiplied by 2 to account for the fact that these movements occur at approximately 2 Hz. Overall variability was the sum of these two components. The components are weakly negatively correlated, indicating that both need to be taken into account in order to gain an overall picture of the rate of change of the face.

### (e) Measuring social relationships

We used available data on social interactions between April 2018 and April 2019—from eight months before video collection started until the end of video clip collection—to construct a dominance hierarchy for both groups. The types of interaction used were displacement, unidirectional aggression, and submission. Each interaction was assigned a winner and a loser: for displacement, the displaced individual was the loser, for unidirectional aggression the aggressive individual was the winner, and for submission the submissive individual was the loser. We used these data to calculate Elo ratings [40,41], with initial ratings set at 1000 with a *k*-value of 50, and only involving interactions between individuals of the same sex (*n* = 182 interactions). Since 63 interactions between males and females were won by males, and none were won by females, Elo ratings of females were recalibrated so that the rating of the dominant female was lower than that of the lowest-ranked male (following [31]), using the formula

$$E_{\mathrm{new}}=\frac{E_{\mathrm{old}}\;(M_{\min}-1)}{F_{\max}},$$

where *E*_old_ is the raw Elo rating of an individual, *E*_new_ is the recalibrated rating, *M*_min_ is the rating of the lowest-ranked male and *F*_max_ is the rating of the highest-ranked female. We calculated the difference in dominance ranking as

$$Elo_{\mathrm{difference}}=Elo_{\mathrm{signaler}}\;\text{–}\;Elo_{\mathrm{receiver}}.$$

If signallers are higher-ranked than receivers we see positive values for Elo rating difference, while if receivers are higher-ranked than signallers we see negative values.

Using the same dataset of social interactions, we also calculated a composite sociality index (*CSI*; [42]), using both focal and scan data, giving an estimate of the strength of social bond between two individuals. The formula used (following [31]) was based on grooming frequency and duration, time spent in proximity (less than 1 m), and frequency of affiliative contact (touching, embracing, etc.). Time in proximity was recorded in scans, while the other components were recorded in all-occurrence sampling. We calculated the *CSI* using the equation

$$CSI=\;\frac{\left(G_{\mathrm{ab}}/G_{x}+D_{\mathrm{ab}}/D_{x}+P_{\mathrm{ab}}/P_{x}+A_{\mathrm{ab}}/A_{x}\right)}{4},$$

where *G*_ab_ is the *frequency* of grooms between individuals a and b, *D*_ab_ is the *duration* of grooms between a and b, *P*_ab_ is the rate of proximity within 1 m and *A*_ab_ is the frequency of affiliative contact. The denominators *G_x_ D_x_ P_x_* and *A_x_* are the overall rates of these in group *x* to which a and b belong. Since individuals were followed for different lengths of time overall, these eight terms were each corrected for sampling effort.

### (f) Statistical analyses

We used the ‘lme4’ package (v. 1.1-26, [43]) in R (v. 4.0.3, [44]) to fit linear mixed-effects models for each dependent variable using maximum-likelihood. Outcome category, sex combination, sociality index and Elo rating difference were included as fixed effects. Elo rating difference was included as both linear and quadratic terms, as our predictions allow for greater intensity at zero values of this variable than at extreme negative or positive values. The dependent variable Intensity was log-transformed as this removed the skew from the residuals; no such transformation was necessary for variability. The only random effect included in our final models was signaller ID. While ideally we would also include receiver ID, and nest both receiver ID and signaller ID within group, this error structure was too complex for the data, leading to no reduction in the residual variance of the model.

We built 41 models for each dependent variable (DV), with all possible combinations of interactions except for those involving the interaction of behavioural outcome and sex combination, since these combinations of two categorical variables created heavily skewed interaction sample sizes. We selected the most parsimonious model for each DV using corrected Akaike's Information Criterion (AICc [45]) and compared this model to the null model using the ‘anova’ function in the ‘lmerTest’ package (v. 3.1-3 [46]). Likelihood ratio tests to assess significance of effects used Satterthwaite's method for approximating degrees of freedom [47], which has been shown to be a reliable method for significance testing in linear mixed models, achieving low rates of type I error even at low sample sizes [48]. To perform pairwise comparisons of categorical predictor variables we employed estimated marginal means with Tukey adjustment via the ‘emmeans’ package (v. 1.6.0 [49]).

## 3. Results

### (a) Intensity

The most parsimonious model by AICc included no interaction terms and was a significantly better fit for the data than the null model (likelihood-ratio test, *χ*^2^ = 68.45, d.f. = 10, *p* < 0.001).

**Figure 1.**
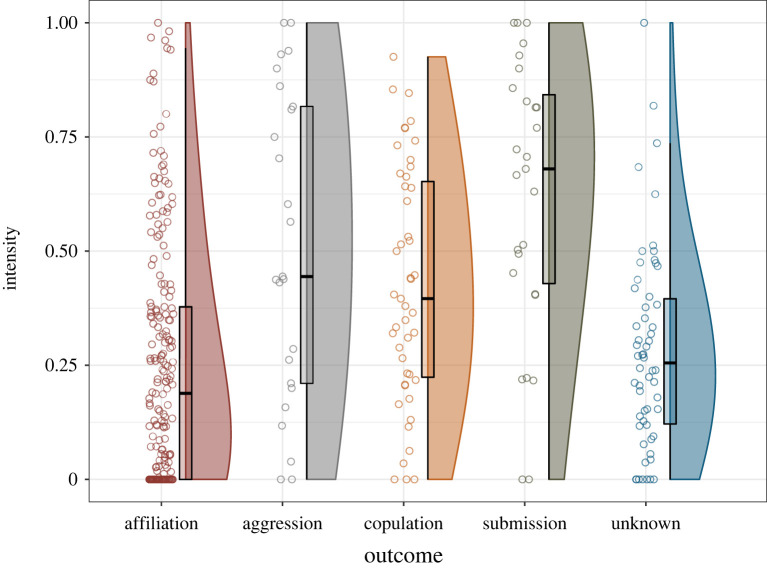
Intensity of facial movements associated with different behavioural categories; ‘unknown’ indicates that the interaction did not clearly belong to any defined category. (Online version in colour.)

**Figure 2.**
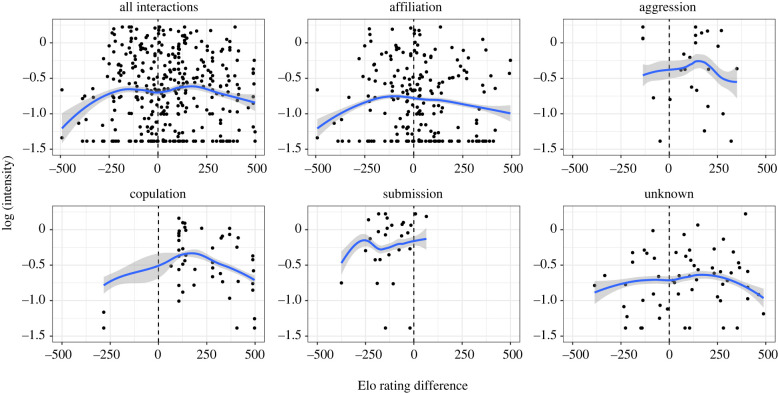
Effect of Elo rating difference on intensity of facial movements. Top-left graph shows the overall trend across all interaction outcome categories. Other plots are for specific interaction outcomes as stated above each plot. Overall best fit lines are based on predicted values from the overall model. Grey shading shows 95% CI for the model; 'unknown' indicates that the interaction did not clearly belong to any defined category. (Online version in colour.)

There was a significant main effect of interaction outcome: affiliative outcomes were associated with lower intensity of facial movements than aggression, copulation or submission outcomes, and aggression and submission interactions were associated with higher intensity than interactions with unknown outcomes, but there were no differences in intensity between facial movements associated with submission, copulation and aggression (table 2 and figure 1). A significant main effect of Elo rating difference demonstrated a negative quadratic relationship: when animals had similar Elo ratings, they tended to use more intense facial movements than when they were less closely matched (figure 2). There was no significant linear component to the effect of Elo rating difference; this relationship was marginally nonsignificant. *CSI* and sex combination also had no significant effect on intensity (table 2).

**Table 2.** Output of linear mixed model for intensity of facial movements (categorical variables split by level).

predictor	estimate	s.e.	d.f.	*t*	*p*
(intercept)	−0.798	0.057	59.41	−13.98	<0.001***
Elo rating difference	−1.470	0.895	157.23	−1.64	0.102
Elo rating difference^2^	−1.412	0.633	243.82	−2.23	0.027*
*CSI*	−0.019	0.013	363.50	−1.43	0.154
pairwise comparisons of behavioural outcome					
affiliation–aggression	−0.402	0.103	361	−3.89	0.001**
affiliation–copulation	−0.342	0.090	363	−3.79	0.002**
affiliation–submission	−0.583	0.097	361	−5.98	<0.001***
affiliation–unknown	−0.084	0.073	359	−1.15	0.779
aggression–copulation	0.060	0.123	364	0.49	0.989
aggression–submission	−0.181	0.132	357	−1.37	0.648
aggression–unknown	0.318	0.112	361	2.85	0.037*
copulation–submission	−0.240	0.127	364	−1.89	0.325
copulation–unknown	0.259	0.097	362	2.68	0.059
submission–unknown	0.499	0.110	361	4.55	<0.001***
pairwise comparisons of sex combination (signaller first)					
♀♀–♀♂	0.184	0.137	347.3	1.34	0.539
♀♀–♂♀	−0.130	0.110	83.3	−1.19	0.637
♀♀–♂♂	0.075	0.097	101.2	0.77	0.867
♀♂–♂♀	−0.314	0.178	185.3	−1.77	0.293
♀♂–♂♂	−0.109	0.156	213.6	−0.70	0.897
♂♀–♂♂	0.205	0.111	341.9	1.85	0.254

***p < 0.001; **p < 0.01; *p < 0.05.

Elo rating difference^2^ = quadratic effect of Elo rating difference.

### (b) Within-interaction variability

The most parsimonious model fit the data significantly better than the null model (likelihood-ratio test, *χ*^2^ = 69.1, d.f. = 10, *p* < 0.001). Effects identified in this model are shown in table 3.

**Table 3.** Output of linear mixed model for variability of facial movements (categorical variables split by level).

predictor	estimate	s.e.	d.f.	*t*	*p*
(intercept)	0.621	0.032	364.0	19.42	<0.001***
*CSI*	−0.014	0.009	364.0	−1.49	0.138
Elo rating difference	−1.560	0.5	364.0	−2.962	0.003**
Elo rating difference^2^	0.882	0.4	364.0	2.254	0.025*
pairwise comparisons of behavioural outcome					
affiliation–aggression	0.212	0.071	364	2.98	0.026*
affiliation–copulation	0.021	0.061	364	0.35	0.997
affiliation–submission	0.073	0.067	364	1.09	0.814
affiliation–unknown	0.172	0.050	364	3.43	0.006**
aggression–copulation	−0.191	0.083	364	−2.29	0.150
aggression–submission	−0.139	0.092	364	−1.52	0.551
aggression–unknown	−0.040	0.077	364	−0.52	0.985
copulation–submission	0.052	0.087	364	0.60	0.976
copulation–unknown	0.151	0.066	364	2.28	0.155
submission–unknown	0.099	0.076	364	1.30	0.689
pairwise comparisons of sex combination (signaller first)					
♀♀–♀♂	0.396	0.091	364.0	4.36	<0.001***
♀♀–♂♀	0.170	0.062	364.0	2.74	0.033*
♀♀–♂♂	0.080	0.058	364.0	1.37	0.517
♀♂–♂♀	−0.226	0.109	364.0	−2.08	0.163
♀♂–♂♂	−0.316	0.098	364.0	−3.22	0.008**
♂♀–♂♂	−0.090	0.074	364.0	−1.22	0.614

***p < 0.001; **p < 0.01; *p < 0.05.

Elo rating difference^2^ = quadratic effect of Elo rating difference.

**Figure 3.**
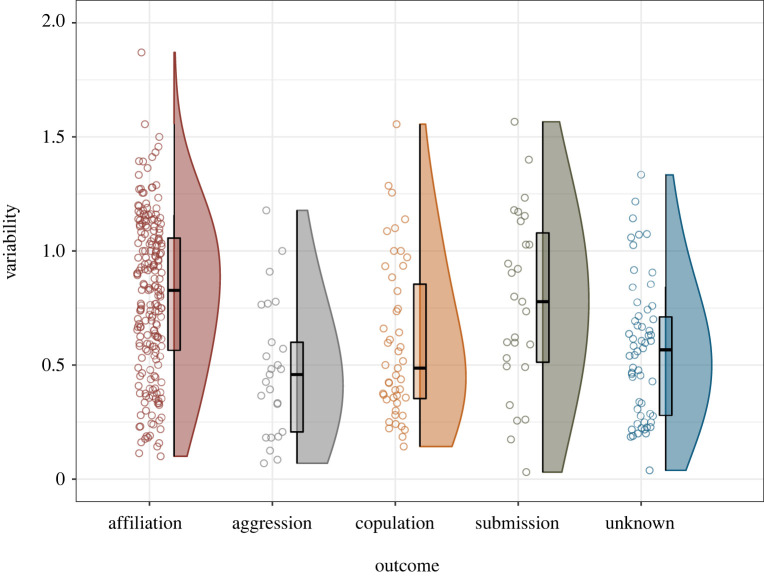
Within-bout variability in facial movements associated with different behavioural categories; ‘unknown’ indicates that the interaction did not clearly belong to any defined category. (Online version in colour.)

**Figure 4.**
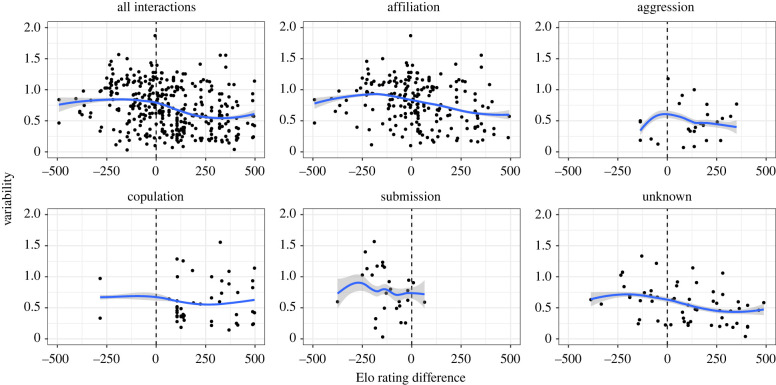
Effect of difference in Elo rating on the variability of facial movements produced, from all interactions. Top-left panel shows the overall trend across all interaction outcome categories. Other panels show the effect for specific social interaction outcomes (indicated above the panels). Best fit lines are based on predicted values from the overall model. Grey shading shows 95% CI for the model; 'unknown' indicates that the interaction did not clearly belong to any defined category. (Online version in colour.)

Affiliation was associated with the greatest variability overall, with significantly greater variability than aggressive interactions or those with unknown outcomes; however, no other significant differences in variability were identified (table 3 and figure 3). Elo rating difference had a significant effect on variability, with displays directed up the hierarchy having greater variability (figure 4). The quadratic term for difference in Elo rating had a significant positive effect, but the effect size was small in comparison to the effect on intensity outlined above (table 2 and figure 2), and the best fit lines appear to be reasonably approximated as linear (figure 4). Sex combination had a significant effect on variability, with same-sex interactions generally involving greater variability of facial movements than those involving individuals of different sexes. Facial movements in female–female interactions were significantly more variable than in male–female and female–male interactions, but not significantly different from those produced in male–male interactions (table 3); male–male interactions involved significantly more variable facial movements than female–male interactions. *CSI* had no effect on variability of facial movements (table 3).

## 4. Discussion

Our main findings were (1) that intensity of facial movements differed according to the type of behavioural interaction they were involved in, with affiliative interactions being associated with significantly lower intensity than aggression, copulation and submission; (2) that variability of facial movements was less affected by the type of behavioural interaction, with the only significant effects being greater variability in affiliative interactions compared to (i) aggressive interactions and (ii) interactions with unknown outcomes; (3) that dominance interactions had some effect on these characteristics, with higher-intensity facial movements being directed to more closely matched individuals, and more variable displays being directed to higher-ranked individuals; (4) that sex combination had no effect on intensity, but that intrasexual interactions involved greater variability of movements than interactions between individuals of different sexes; and (5) that the strength of social bonds had no discernible effect on either intensity or variability.

### (a) Effects of behavioural context

Theoretically, potentially more valuable or costly interactions should involve more exaggerated signals to reduce ambiguity. This should mean that intensity of signals is higher in aggressive and submissive contexts, and potentially in copulation contexts as well since these situations have relatively high fitness costs. We also expected reduced variability in these contexts, since variability might lead to ambiguity of signals (although see §4d below). Our results support these predictions, with affiliative outcomes being associated with the least intense and most variable facial movements. Submission, copulation and aggression outcomes were associated with movements of greater intensity than affiliation; while differences in variability were less pronounced, aggression outcomes were associated with significantly reduced variability.

Our findings reinforce those of our earlier study of bared-teeth displays (BT; [17]) that found higher rates of variability in affiliative BT than copulation, play and submission BT. It is possible that the greater variability observed in affiliative displays is due to the reduction in selective pressure in this situation, meaning that facial movements are less tightly constrained. It should be noted that the present study measured variability differently: dynamic displays such as teeth chattering, lipsmacking and jaw wobbling are counted toward variability in this study but were not in the previous study. Since lipsmacking is generally an affiliative signal in crested macaques [19,31], teeth chattering is submissive [17] and jaw wobbling is used by males to solicit mating [17,19], this new measure of variability will have produced elevated values in these behavioural categories. Increased variability of facial movements may be produced either by repetitive dynamic displays (i.e. predictable movements) or by unpredictable, unritualized movements. While each of these aspects of variability may act to increase conspicuousness, arguably the presence of repetitive dynamic displays could reduce uncertainty whereas unpredictable, unritualized movements may increase uncertainty as to the information content of the signal.

### (b) Effects of dominance relationship

Our measure of dominance relationships was the difference in Elo rating between the signaller and the receiver, which reflects the direction and magnitude of power differences within a dyad.

Greater intensity of facial movements occurred when power differences between individuals were low, while there was no trend of greater intensity in displays being directed up or down the hierarchy. Social interactions between female crested macaques are characterized by moderately skewed dominance relationships and frequent, low-level conflict that is often bidirectional [50,51], though the picture is less clear for male–male interactions and for those between sexes. Thus, crested macaque social interactions are perhaps less predictable than in other macaques where dominance relationships are more rigid and aggression is more often unidirectional, as in rhesus (*Macaca mulatta*) and Japanese macaques (*M. fuscata*) [33]. The highest likelihood of misunderstanding is in closely matched pairs, so it is possible that crested macaques use more intense signals in these situations to reduce the potential for ambiguity and misunderstandings. The proximate reasons for increased intensity are unknown: animals may produce intense vocalizations or movements intentionally in important or uncertain situations in order to ensure that their message is received and understood, or they may do so unintentionally because of some aspect of internal state, such as experiencing elevated arousal in these situations [52].

Elo rating difference also had a significant effect on the variability of facial movements, with increased variability of movements when the signaller had a lower Elo rating than the receiver. This may indicate that affiliative interactions are not symmetrical in crested macaques, with different types of display being used for appeasement by lower-ranked individuals and reassurance by higher-ranked individuals. Since the silent bared-teeth, a relatively static facial display, has been purported to be a signal of benign intent in other species (e.g. mandrills [53], moor macaques [54]), this may be an example of a relatively static reassurance signal, whereas dynamic displays may be more often used by subordinate animals seeking to appease dominant ones. Prior studies of crested macaques support this idea to some extent, with dynamic displays such as teeth-chattering being linked to submission [17]. Lipsmacking is also used in general to start affiliative interactions [31], as well as being an often-used signal in reconciliation in this species [55]. Though lipsmacking is often reciprocated during social interactions, subordinates are more likely to reciprocate lipsmacks of dominants than *vice versa* (P. Clark 2021, unpublished data), which fits with the observed tendency for more variable displays to be directed up the hierarchy more often than down.

### (c) Relationship quality

We predicted that increased social bond strength, measured by a composite sociality index (*CSI*), would reduce the intensity and increase the variability of facial movements since ambiguity should be less confusing and perhaps less costly between animals with a closer social relationship. However, we found no evidence for any significant overall effect of *CSI* on either intensity or variability. It appears that having a stronger social bond does not mitigate the need for increased intensity of movements in certain contexts, nor liberate facial displays into greater overall variability. This finding that individuals don't change their communication style based on social bond strength is particularly interesting in light of the contrast with the effect of dominance relationship contrasts, indicating that dominance, rather than friendship, appears to drive the flexible use of facial communication in crested macaques. This finding contrasts with previous work that demonstrated effects of social bonds on communicative signals in this species [31,56,57].

### (d) Measuring variability

Measuring variability poses a problem for this type of study, in the same way that measuring the complexity of communication systems is problematic [58]: there are a number of ways to define and measure variability. In this study, we have treated variability as a composite of changes in AU combination, changes in AU intensity and an estimate of the frequency of appearance changes visible during dynamic ritualized facial displays. The aim of this strategy was to gain an overall measure of the rate at which the appearance of the face changes. However, we outlined earlier that crested macaques exhibit several dynamic ritualised facial movement combinations, including lipsmacking, teeth-chattering etc. It could be argued that ritualized movements reduce rather than increase unpredictability, as the appearance of the face oscillates between two configurations; contrast this with unritualized movements where any possible configuration is possible. It is possible that the variability provided by dynamic ritualized movements plays a similar role to intensity, being conspicuous and exaggerated in order to avoid ambiguity. Under this framework, unpredictable and non-ritualized movements would be expected in situations where ambiguity is less costly. This type of variability would not be expected between individuals in a close social relationship. The measure of variability in this study, however, has been chosen because it requires less-subjective decisions on the researcher's part: we have simply attempted to approximate the rate at which the appearance of the face changes, without assigning any value in terms of the meaning that a receiver can obtain.

## 5. Conclusion

This study provides a fine-tuned analysis of how communication can consolidate social relationships. We demonstrated differences in intensity and variability of facial movements used in different contexts. We found that dominance ranking differences have some effect on these characteristics of facial communication, but no evidence for any impact of social relationship quality. Our findings support the idea that a function of intensity in communicative signals is to reduce ambiguity in situations where confusion is more likely and potentially more costly, both in terms of the immediate context of a single social interaction and the long-term context of a dominance relationship. Contrary to our predictions we found no mitigating effect of social relationship closeness, indicating that dominance, rather than the strength of a social bond, has the greatest impact on communication style.

Our finding that crested macaques use facial movements of greater intensity in riskier situations indicates that production of subtly different facial signals may be important for navigating social interactions, and consequently for the formation and maintenance of social bonds. This supports the social complexity hypothesis for the evolution of complex communication [35], but has implications for how we measure and quantify facial expressions. Complexity in facial communication is sometimes thought of as the number of discrete expressions that a species produces (e.g. [20,21]), but this overlooks the potential added complexity of graded expressions where differences in intensity might affect function. While many studies attempt to tie facial signals to their functions, we have illustrated that social signals can be used differently depending on the context of both the immediate social interaction and the long-term social relationship. By conducting analysis at fine temporal and spatial scales we have illustrated that variability in the social world could favour the graded use of facial signals, thereby increasing the complexity of the communication system.

Overall, these results indicate that signal intensity and variability may be important factors in enabling individuals to navigate varied social interactions. It would be beneficial to examine this potential link more directly in future; studies investigating the function of specific facial movements could consider the intensity of those movements, and studies examining the complexity of facial communication should also take into account the graded and dynamic nature of facial movements.

## Data Availability

Data and code are available online at Figshare [59]. The data are provided in electronic supplementary material [60].
